# Supplementary material for: Risk of Major Cardiovascular Disease after Exposure to Contrast Media: A Nationwide Population-Based Cohort Study on Dialysis Patients
Source: Metabolites. 2023 Feb 13;13(2):266. doi: 10.3390/metabo13020266 (PMC9959650; doi:10.3390/metabo13020266)
Supplement: Supplementary file 1 [file metabolites-13-00266-s001.zip › metabolites-2098632-supplementary.pdf]

Supplement Table S1. ICD-9 and ICD-10 codes applied to inclusion criteria, outcome events, and comorbidities.

Item	ICD-9	ICD-10
**Inclusion criteria**		
End-stage renal disease	585	N18.6
**Outcome**		
Major adverse cardiovascular events (MACE)	410, 411, 427.5, 798.1, 798.2, 428, 434-436, 437.1	I20-I22, , I46, I50, I63
Acute coronary syndrome(ACS) /Acute Myocardial Infarction(AMI)	410, 411	I20-I22
Sudden cardiac arrest (SCA)	4275, 798.1, 798.2	I46
Heart failure	428	I50
Stroke	430.x-438.x	G45-G46, I60-I66, I68, I69
**Comorbidities**		
Hypertension	401–405	I10-I16
Diabetic	250.x	E10, E11
Hyperlipidemia	272.x (excluding 272.5x, 272.8)	E78
Dysrhythmia	427	I47.0, I47.1, I47.2, I48.0, I48.2, I48.91, I49.1, I49.2, I49.3, I49.0
Chronic liver disease	571, 572.2-572.8 , 573.1-573.3, 456.0-456.2	B15.0, B16.0, B16.2, B19.0, K70.4, K72, K76.6, I85
Chronic obstructive pulmonary disease (COPD)	490–492, 496	J40-J44

Supplement Table S2. Oral prescription medications analyzed in the study.

Drug classification	ATC code
Hypoglycemia agents	A10B (excluding A10BJ)
Angiotensin Converting Enzyme inhibitors; ARB, Angiotensin receptor blockers	C09, C10BX
Beta blocker	C07(excluding C07AA05), C09BX, C09DX
Calcium channel blockers	C08G, C09BB, C07FB, C10BX
Thiazide diuretics	C03, C07B, C07C, C07D ,C08G, C09BA ,C09DA
Statin	C10AA, C10B
NSAID	M01A
